# Cholinergic basal forebrain neurons regulate fear extinction consolidation through p75 neurotrophin receptor signaling

**DOI:** 10.1038/s41398-018-0248-x

**Published:** 2018-09-21

**Authors:** Zoran Boskovic, Michael R. Milne, Lei Qian, Hamish D. Clifton, Alice E. McGovern, Marion T. Turnbull, Stuart B. Mazzone, Elizabeth J. Coulson

**Affiliations:** 1 Centre for Ageing Dementia Research, The University of Queensland, Brisbane, QLD 4072 Australia; 2 Queensland Brain Institute, The University of Queensland, Brisbane, QLD 4072 Australia; 3 Faculty of Medicine, School of Biomedical Sciences, The University of Queensland, Brisbane, QLD 4072 Australia; 4 Department of Pharmacology, University of Oxford, Oxford, UK

## Abstract

Cholinergic basal forebrain (cBF)-derived neurotransmission plays a crucial role in regulating neuronal function throughout the cortex, yet the mechanisms controlling cholinergic innervation to downstream targets have not been elucidated. Here we report that removing the p75 neurotrophin receptor (p75^NTR^) from cBF neurons induces a significant impairment in fear extinction consolidation. We demonstrate that this is achieved through alterations in synaptic connectivity and functional activity within the medial prefrontal cortex. These deficits revert back to wild-type levels upon re-expression of the active domain of p75^NTR^ in adult animals. These findings demonstrate a novel role for cholinergic neurons in fear extinction consolidation and suggest that neurotrophic signaling is a key regulator of cholinergic-cortical innervation and function.

## Introduction

Cholinergic basal forebrain (cBF) neurons play an important role in cognition through their modulation of cortical neurotransmission^1,2^. These neurons have a significant influence on the activity of neuronal populations within a variety of cortical and subcortical areas, including the entorhinal cortex^3–5^, neocortex^6^, visual cortex^7^, striatum^8^, hippocampus^9^ and motor cortex^10,11^, with cholinergic dysfunction underpining cognitive impairment in a number of neurodegenerative and mental health conditions^12–14^.

One of the defining features of cBF neurons is that they comprise one of the few populations in the adult brain that expresses the p75 neurotrophin receptor (p75^NTR^). We and others have shown that mice that completely lack p75^NTR^ from conception or those in which p75^NTR^ expression is removed from cBF neurons from postnatal day 4 have altered cBF neuronal innervation to the cortex, which correlates with improvements in spatial navigation, indicative of enhanced memory^15–19^. These data suggested that the function of cortical neurons is regulated by the extent of cBF innervation to target regions, which is mediated through the actions of p75^NTR^ signaling. However, it is unclear whether p75^NTR^ plays a role in axonal innervation and cortical function during adult life rather than development.

Recently, it has been suggested that cBF neurons are involved in regulating the response to aversive stimuli, with fear conditioning being regulated by nucleus basalis of Meynert cBF innervation of the amygdala, and lesionining of medial septal cBF neurons impairing the acquisition of fear extinction—the process whereby fearful responses can be diminished^20–22^. Compared with spatial navigation, the neural circuits and behavioral paradigms associated with fear conditioning and extinction are more clearly defined, allowing cBF neuronal function to be interrogated. In fear extinction paradigms, a conditioned stimulus (CS), such as a tone, is paired with an aversive unconditioned stimulus (US) such as a footshock, leading to a long-lasting response to the CS^23^. This response is diminished through fear extinction by subsequent repeated CS presentations that are not paired with the US. While the hippocampus provides context encoding for fear learning and extinction^24^, it is the infralimbic prefrontal cortex (ILPFC) within the medial prefrontal cortex (mPFC) that is critical for fear extinction consolidation^25,26^.

We hypothesized that, if p75^NTR^ played a role in adult cBF connectivity and function, we would observe a phenotype in adult conditional p75^NTR^ knockout mice (ChAT-cre p75^in/in^), which would be rescued by acute re-expression of p75^NTR^ within cBF neurons. We therefore tested these mice in fear conditioning/extinction paradigms and measured the cBF axonal and synaptic arborization within the relevant circuit.

## Materials and methods

### Animals

The p75^fl/fl^ conditional knockout mouse^16^ and the choline-acetyltransferase (ChAT)-IRES-cre strain^27^ have been described previously. All animals used were 2–4 months of age. All behavioral studies were performed using male mice. Neuronal tracing studies were performed using both males and females. Mice were maintained on a 12-hour light/dark cycle (lights on at 7:00 a.m.), with food and water provided ad libitum in specific pathogen free (SPF) OptiMouse caging. All procedures were approved by the University of Queensland Animal Ethics Committee and conducted in accordance with the Australian Code of Practice for the Care and Use of Animals for Scientific Purposes.

### Behavioral tasks

**Fig. 1:**
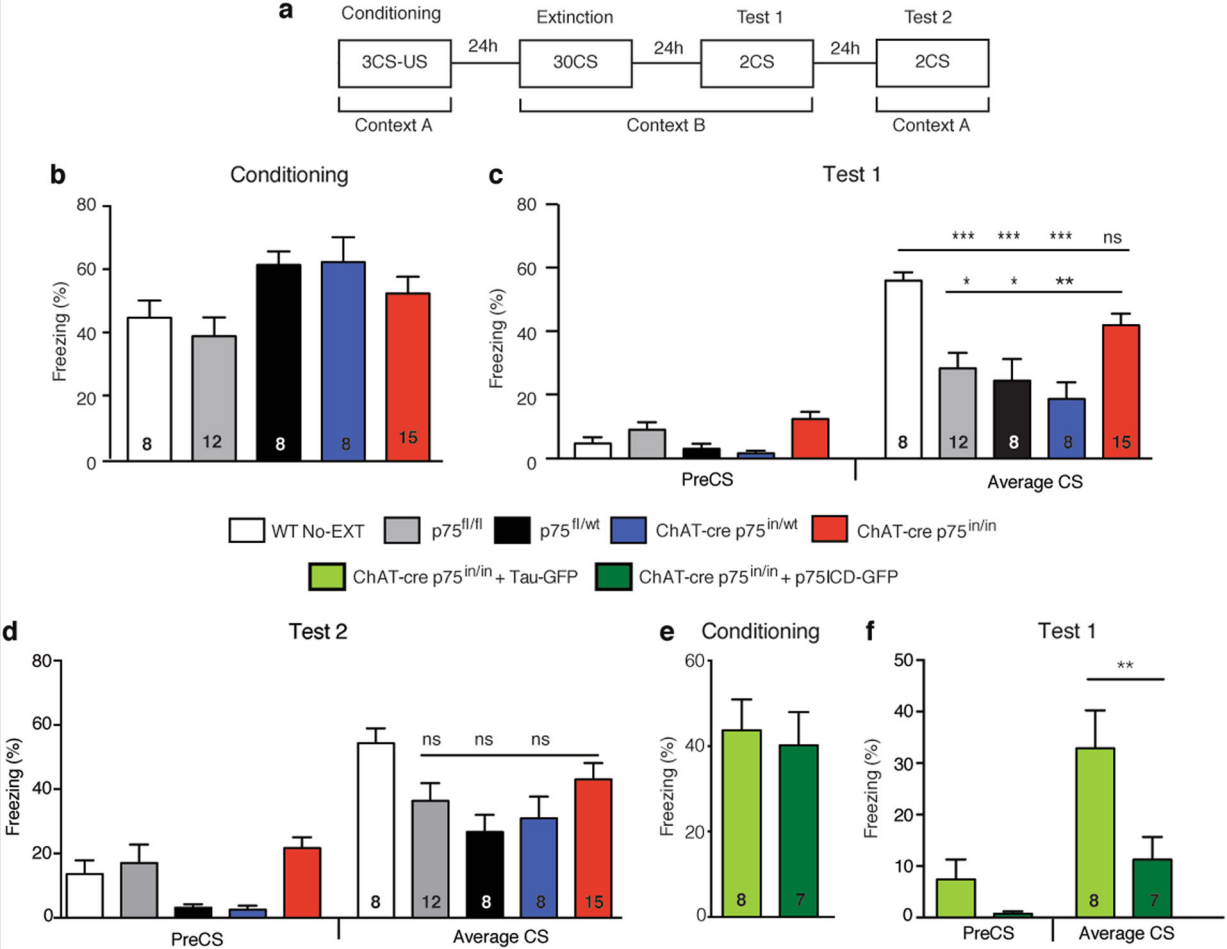
Fear extinction consolidation is impaired in p75^NTR^-deficient animals. **a** The experimental paradigm for assessing fear extinction consolidation consisted of a four-day protocol. On the first day (Conditioning) animals were conditioned by pairing 3 conditioned stimuli (CS) to a unconditioned stimulus (US) in context A. On the second day (Extinction) all animals, were moved to a different context (context B) and, except for the No-EXT group, exposed to 30 CS without a US pairing. Twenty-four hours later, fear extinction consolidation was tested in context B (Test 1) with 2 CS presentations. On the final day, animals were returned to context A and exposed to 2 CS presentations to test context discrimination (Test 2). **b** Average fear response of control (WT No-EXT = wild-type animals that did not undergo extinction training; p75^fl/fl^ = transgenic controls; p75^fl/wt^ = transgenic heterozygous controls; ChAT-cre p75^in/wt^ = heterozygous mutants) and mutant (ChAT-cre p75^in/in^) animals during conditioning training. There were no significant differences in conditioning between any of the groups. **c** Average fear response of animals on day 3 of behavioral testing. Fear extinction consolidation was assessed prior to the first CS presentation (PreCS) and as an average freezing time during the 2 CS presentations (Average CS). All mice displayed significant freezing on CS presentation compared to PreCS levels. Average CS assessment revealed that ChAT-cre p75^in/in^ animals displayed significantly higher freezing than transgenic control groups (two-way ANOVA comparing preCS to CS as well as genotype, *p < 0.05; **p < 0.01, ***p < 0.001) but their degree of freezing was not significantly different from the WT No-EXT control). Data are represented as mean±SEM). **d** Fear assessment of mice when they were returned to the conditioning context on day 4 of behavioral testing. There were no significant differences between any of the transgenic groups, indicating that context discrimination was intact in all of these mice. **e** Average freezing response of ChAT-cre p75^in/in^ mice that received control (Tau-GFP) and p75ICD-GFP virus. There was no significant difference between the two groups during conditioning. **f** Average freezing response of injected ChAT-cre p75^in/in^ mice during fear extinction consolidation testing. Mutant mice that received p75ICD-GFP showed significantly less freezing during the Average CS period compared to Tau-GFP-injected mutants (two-way ANOVA, **p < 0.01; data are represented as mean±SEM). The number of animals analyzed is indicated in the graphs

Fear extinction was assessed using a previously described protocol^26^ (Fig. 1a). Two contexts (A and B) were used. Both conditioning chambers (Coulbourn Instruments) had two transparent walls and stainless steel grid floors (3.2 mm in diameter, 8 mm appart); however, the floors in context B were covered with flat white acrylic inserts to minimize context generalization. Context A had a lemon-scented odor and context B had a vinegar-scented odor. Individual digital cameras were mounted in the ceiling of each chamber and connected via a quad processor for automated scoring of freezing (Freezeframe). Fear conditioning was induced in context A. Mice received 3 CS/US pairings at equal intervals over 14 min. The CS was a 120 s white noise stimulus at 80 dB whereas the US was a 0.5 mA, 1 s footshock. Twenty-four hours later, animals underwent extinction training in context B over 65 min. A 120 s PreCS period was followed by 30 unpaired CS of 120 s with 5 s between tones finishing with 120 s of silence. Mice that did not undergo extinction training were placed in context B but did not receive any CS presentations.The extinction test was a further 24 h later in context B, where they were exposed to two unpaired CS. Finally, to measure contextual fear memory animals were returned to context A, where they were exposed to 2 unpaired CS. Animals with freezing below 10 percent on the conditioning day were excluded from the analysis. The data for freezing prior to the first CS (PreCS) and the average freezing during both CS presentations (Average CS) during testing are provided in the results.

### Viruses

Tau-GFP (used to assess cholinergic innervation) and p75^NTR^ intracellular domain-green fluorescent fusion protein (p75ICD-GFP; used for rescue experiments) expression constructs were packaged into a pseudotype adeno-associated virus (AAV) 2/1 chimeric vector for in vivo delivery into the mice. Each cDNA was inserted into the DIO Cre-ON plasmid in reverse orientation, which was driven by the ubiquitous promoter elongation factor-1 alpha (EF1α) and was flanked by oppositely oriented loxP and lox2272 sites (pAAV-EF1a-DIO). In the presence of cre recombinase, the transgene was expressed^28^. The AAV pseudotyped vectors were generated as previously described^29^. The trans-synaptic HSV129 tracer virus has also been described previously^30^.

### Surgical procedures

Animals were anesthetized by intraperitoneal (i.p.) injection of ketamine (100 mg/kg) and the muscle relaxant xylazine (10 mg/kg). Each mouse was then placed in a stereotaxic frame (David Kopf Instruments) and the skull exposed. Viral infusions were performed using a 30 G infusing system using a guide and internal cannula (Plastics1), directly into the medial septum (the needle was lowered into the medial septum (A–P 0.9 mm; M–L 0 mm; D–V 4.2 mm from Bregma) where either undiluted HSV129 virus (0.5 µl), Tau-GFP (1 µl) or p75ICD-GFP (1 µl) was infused. For all procedures, the internal cannula was left in place for 8 min to allow for diffusion before removal. Immediately after surgery, mice were injected subcutaneously with the analgesic torbugesic (2 mg/kg), and the antibiotic Baytril (5 mg/kg) and allowed to recover before being returned to their home cage where they were housed two per cage but kept separated by a clear Perspex barrier that allowed aural and olfactory exchange. Mice were 8–12 weeks old at the time of injection and were left for 1 month (Tau-GFP and p75ICD-GFP) or 48–72 h (HSV129) to allow for viral expression before behavioral testing/killing.

### Histological tissue preparation

Brains from 4% formaldehyde-perfused mice were post-fixed overnight, preserved in 20% sucrose solution for 24 h, and embedded in O.C.T compound (Tissue-Tek). Forty µm coronal sections were cut in three serially adjacent sets through the basal forebrain and prefrontal cortex using a sliding microtome (SM2000r, Leica). Brains used for phosphorylated extracellular signal-regulated kinase (pERK) quantification were from naïve mice or those euthanized not more than 1 h after the fear extinction test.

### Immunohistochemistry

For immunofluorescence labeling, free-floating 40 µm sections were immunostained using goat anti-GFP (1:1000, Abcam ab5450), rabbit anti-DsRed (1:200; Clontech 632496), rabbit anti-pERK (1:200; Millipore 05-797 R) and mouse anti-CamKII (1:200; Abcam ab22609). Sections were mounted onto slides and coverslipped using fluorescence mounting medium (Dako S3023).

To measure neuronal activity, labeling of pERK was visualized using rabbit anti-pERK antibody (1:1000; Millipore 05-797 R), biotinylated donkey anti-rabbit IgG (1:1000; Abcam ab97062) and ABC reagent (Vector Elite kit: Vector Laboratories PK-6100). Black immunoreactive cytoplasm labeled for pERK was revealed by a nickel-intensified diaminobenzidine reaction and slices were coverslipped with DePeX (Sigma-Aldrich 06522).

### Histological quantifications

All cell counts and quantifications for the mPFC were performed on sections between 2.3 mm anterior to bregma and 1.5 mm anterior to bregma, medial septum/diagonal band of Broca sections were between 1.3 mm anterior to bregma and 0.1 mm anterior to bregma and hippocampal sections from between 1.2 mm posterior to bregma and 2.2 mm posterior to bregma. Sections from these regions were randomly selected and the researcher was blind to the group allocation. When quantifying subregions of the mPFC, immunohistological image of the section being analyzed was overlayed with the Allen brain atlas demarcations on a computer at a high magnification, with stereological rules being applied when cells were touching a line.

#### Neurite density

To measure cholinergic innervation in the mPFC and hippocampus on a per cell basis, Tau-GFP and p75ICD-GFP virus-infused animals were euthanized following a 6-week incubation period and the extent of GFP labeling was used as a proxy for density of cBF neuronal innervation. First, the number of GFP-positive cell bodies in every third section at the injection site was counted. Then 3-6 mPFC sections were randomly selected from each brain and stained for GFP and imaged. If fewer than 10 cells per section were infected, or there were no obvious GFP-positive axons in the target areas the animals were excluded from the quantification The images were converted to an 8-bit black and white image for analysis using ImageJ v1.49 (NIH). A region of interest (ROI) was drawn around the mPFC/hippocampal area of each section aligned to the atlas of Franklin and Paxinos (2007). Threshold settings were used to subtract background staining and artifacts from the image. The mean pixel density within the ROI was then calculated and averaged for each mouse to provide the average neurite density in the mPFC/hippocampus. These data were then converted to average density per infected neuron at the injected site for each mouse by dividing the neurite density by the number of labeled neurons. The average ratio was compared between genotypes.

#### Synaptic connectivity

To measure the extent of synaptic connectivity from the medial septum to the mPFC and hippocampus, the number of HSV129 GFP- and tdTomato-labeled neurons in the mPFC (6–8 randomly selected sections per animal) and hippocampus (7–10 randomly selected sections per animal) were counted following tissue processing and immunohistochemistry.

#### Imaging

Imaging was performed on an Axio Imager upright fluorescence microscope (Zeiss) and Metafer VSlide Scanner (Metasystems) using Zeiss Axio Imager Z2. All measurements and analyses were performed using Imaris 7.2.3 software (Bitplane).

### Statistics

Results are expressed as mean ± standard error of mean. Statistical analyses were conducted using two-tailed student’s t-tests, one-way ANOVA or two-way ANOVA (CS one factor and genotype second factor) with Sidak’s post-hoc test (Graphpad Prism 6) with the significance threshold set at *p* < 0.05.

## Results

### p75^NTR^ is required for successful fear extinction consolidation

To determine the effects of p75^NTR^ removal from cBF neurons on fear extinction, we subjected two cohorts of ChAT-cre p75^in/in^ mice (mice in which the floxed allele was inverted—in/in—in cholinergic neurons) and littermate heterozygous and cre-negative controls^16^ to a four-day, strong extinction paradigm (Fig. 1a–c). Mice of all genotypes showed similar freezing behavior following conditioning training on day 1(F (4, 46) = 0.756, *p* = 0.5592). Similarly, we observed no difference between groups during extinction training on the following day (data not shown), with all mice showing ~40% freezing on first CS presentation compared to preCS levels (CS factor: F (1, 92) = 132.2; *p* < 0.0001), and freezing decreased over 10 CS presentations (F (10, 275) = 3.305, *p* = 0.0005). However, there was no difference in freezing response between genotypes (time/genotype interaction F (10, 275) = 0.6975, *p* = 0.7267). Conversely, during the extinction test on the third day, although all mice froze significantly on first CS presentation compared to preCS levels (CS factor: F (1, 92) = 132.2; *p* < 0.0001), there was a significant interaction of genotype in response to the CS (F (4, 92) = 5.917, *p* = 0.0003), with p75^NTR^-knockout mice showing significantly greater freezing than cre-negative homozygous (p75^fl/fl^; *p* = 0.036) and heterozygous controls (p75^fl/wt^; *p* = 0.011), as well as heterozygous mutants (ChAT-cre p75^in/wt^; *p* = 0.0002). Furthermore, the freezing behavior of ChAT-cre p75^in/in^ animals was not significantly different from that of wild-type animals which underwent conditioning but not extinction training. Nonetheless, when returned to the original conditioning context on day 4, there were no significant differences in the levels of freezing between ChAT-cre p75^in/in^ animals, littermate p75^fl/fl^ controls and heterozygous controls that had undergone extinction training (Fig. 1d; CS/genotype interaction F (4, 92) = 1.353, *p* = 0.2566). This indicates that the deficit in the p75^NTR^-knockout animals is specific to consolidation of fear extinction rather than any other aspect of fear-related behavior.

Recombination of the p75^NTR^ allele in ChAT-cre p75^in/in^ animals prevents p75^NTR^ expression from postnatal day 4^16^. Hence, the observed behavioral change could be a consequence of differences in postnatal development (reduced programmed cell death resulting in a ~25% increase in adult cBF neurons^16^) or the result of an acute role for p75^NTR^ in adult neurons. The intracellular domain of p75^NTR^ (p75ICD) has been identified as a critical mediator of p75^NTR^ function^31^. This fragment lacks the ligand-binding and transmembrane domains but is considered to be a constitutively activated form of p75^NTR^^32^ as it can mediate both axonal pruning^33^ and axonal growth, in the latter case by facilitating Trk receptor signaling^31,34^. The p75ICD-GFP AAV, or a control AAV containing the identical genetic backbone but expressing a benign GFP-tagged tau protein (Tau-GFP; Supplementary Figure S1a, b), was injected into the medial septum of the basal forebrain in adult ChAT-cre p75^in/in^ animals. Following a 6-week period to allow for robust expression of the virus (97 ± 0.71% of medial septum ChAT-positive cells expressed GFP; data not shown), the AAV-injected mutant animals were subjected to the same fear conditioning/extinction paradigm. Locomotion and exploratory behavior were not affected by p75ICD-GFP expression (Supplementary Figure S1c, d), and no differences were observed during conditioning training (Fig. 1e). However, p75ICD-GFP-injected ChAT-cre p75^in/in^ animals displayed a significant decrease in freezing during the extinction test compared to control-injected ChAT-cre p75^in/in^ animals (Fig. 1f; Treatment factor F (1, 26) = 13.08; *p* = 0.0013), possibly freezing less than p75^fl/fl^ animals (*c.f*. Fig. 1c). These results demonstrate that medial septum cBF neurons are important for regulating the consolidation of fear extinction and that p75^NTR^ signaling in the adult cBF neurons is involved in this process.

### p75^NTR^ has a negligible effect on the extent of innervation of cholinergic neurons to the PFC

**Fig. 2:**
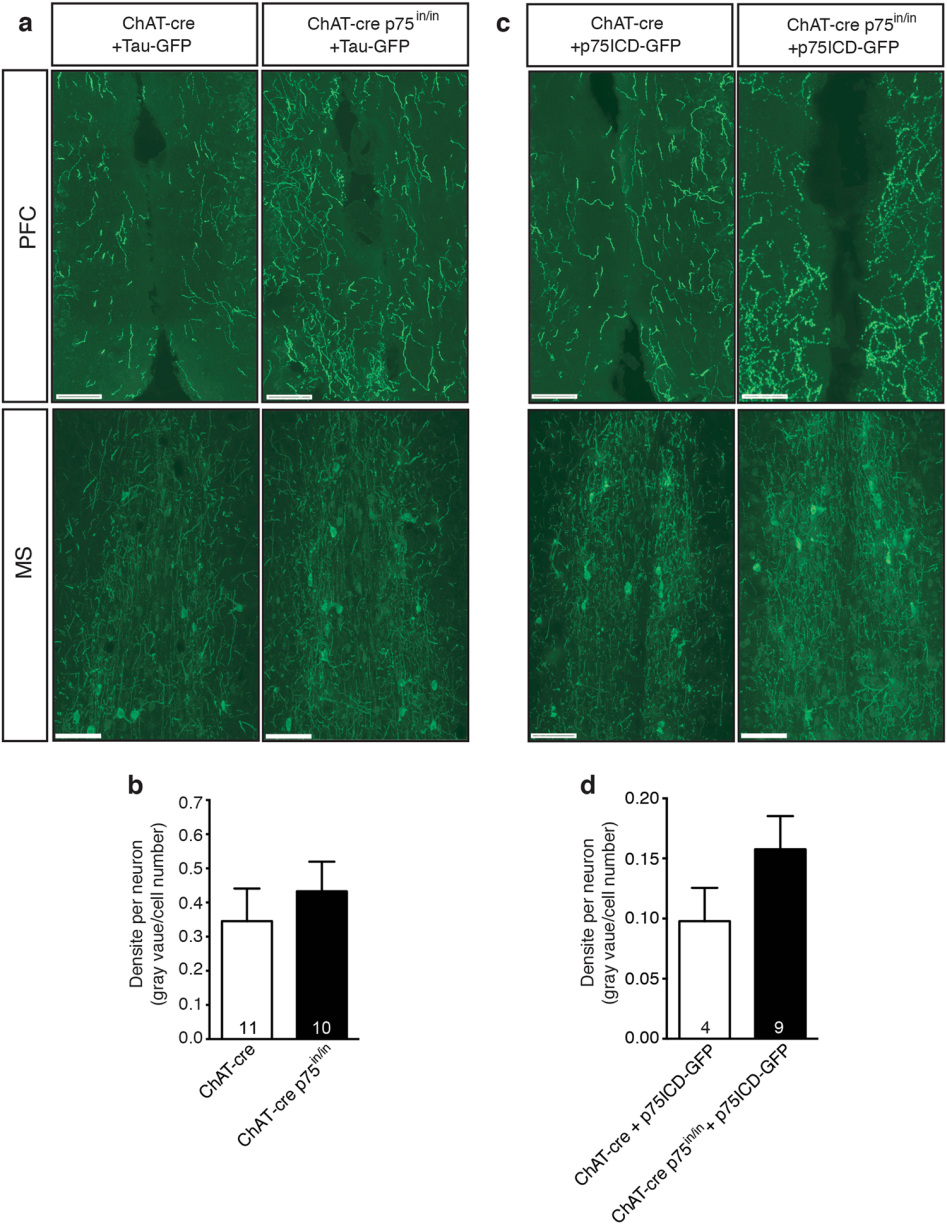
Arbour size of individual neurons is unaffected by the absence of p75^NTR^. **a** Representative images of the prefrontal cortex (PFC; target) and medial septum (MS; injection site) and of cre-expressing control (ChAT-cre) and mutant (ChAT-cre p75^in/in^) mice injected with Tau-GFP. Sections were labeled using an anti-GFP antibody (green). Scale bar = 100 µm. **b** Average GFP-labeled axonal density per infected cell of mice injected with Tau-GFP AAV. No significant difference in axonal density/cBF neuron was observed between control and mutant mice. **c** Representative images of cBF axonal innervation of the PFC and infected cells in the MS of control and mutant mice injected with p75ICD-GFP. Sections were labeled using an anti-GFP antibody (green). **d** Average GFP-labeled axonal density per infected cell of animals injected with p75ICD-GFP AAV. No significant effect of the p75ICD-GFP injections were observed in animals of either genotype. The number of animals analyzed is indicated in the graphs

The observed behavioral changes in ChAT-cre p75^in/in^ animals could be due to alterations in cBF innervation of downstream cortical targets that are involved in fear extinction. We have previously demonstrated that ChAT-cre p75^in/in^ animals have increased cholinergic innervation of the barrel cortex but not the hippocampus^16^. However, we did not determine whether this phenotype was due to the 25% increase in the number of cBF neurons, or whether individual cBF neurons have increased arborization in the absence of p75^NTR^. To investigate this, we injected the cre recombinase-dependent Tau-GFP AAV into the medial septum of control (ChAT-cre) and ChAT-cre p75^in/in^ animals, thereby labeling cBF axons. Brain structures known to be important in fear extinction consolidation include the amygdala, hippocampus and mPFC^1,25,35^. Medial septum cBF neurons project to the latter two regions but not the amygdala, which is innervated by the nucleus basalis of Meynert cBF neurons. We therefore quantified the density of GFP-labeled cBF axons in the hippocampus and mPFC relative to the number of medial septum cBF neurons infected with the virus. No significant difference was observed between mutant and control animals in terms of the extent of GFP labeling in either the hippocampus (Supplementary Figure S2a, b) or the mPFC (Fig. 2a, b).

We also assessed cBF axonal innervation in the PFC and hippocampus in control and ChAT-cre p75^in/in^ mice expressing p75ICD-GFP by quantifying the extent of axonal GFP staining. Expression of p75ICD-GFP in p75^NTR^-deficient cBF neurons had no significant effect on hippocampal (Supplementary Figure S2c, d) or mPFC innervation in comparison to its effect on wild-type cBF neurons (Fig. 2c, d). This indicates that re-expression of p75ICD does not restore fear extinction by mediating axonal pruning.

### p75^NTR^ regulates basal forebrain connectivity to the PFC

**Fig. 3:**
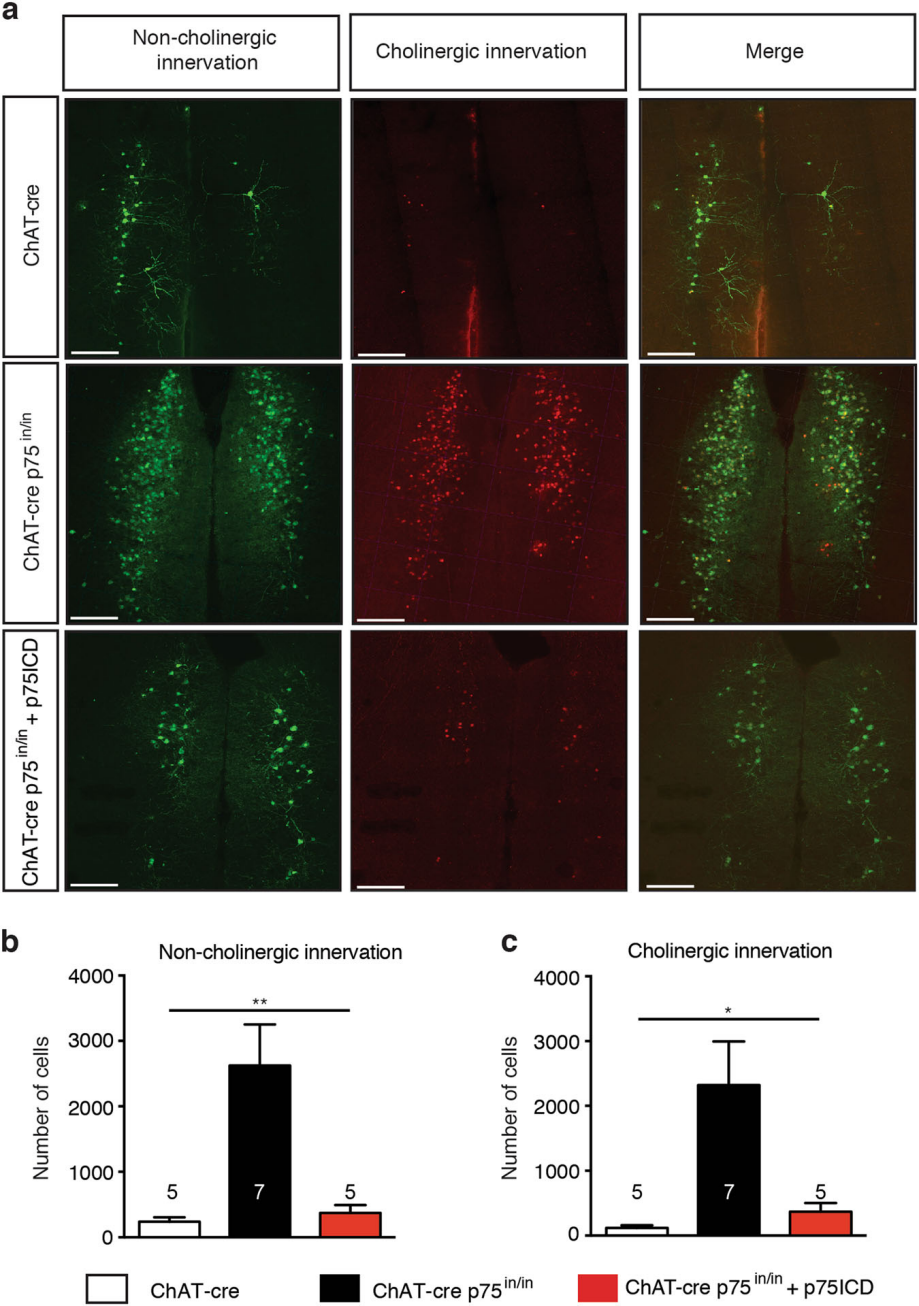
p75^NTR^ signalling affects synaptic connectivity of cBF neurons. **a** Representative images of the prefrontal cortex (PFC) of cre-expressing controls (ChAT-cre), mutants (ChAT-cre p75^in/in^) and mutants injected with p75ICD-GFP. Postsynaptic cells in the PFC innervated by non-cholinergic basal forebrain neurons (green) were labeled against the GFP-expressing version of HSV129 virus (See Supplementary Figure S3A). Cells innervated by cBF neurons (red) were labeled against the tdTomato-expressing version of the HSV129 virus. Scale bar = 200 µm. **b** There was a significant increase in GFP labeling in the PFC of ChAT-cre p75^in/in^ mutants compared to control and p75ICD-injected mutant mice (one-way ANOVA, **p < 0.01; the data are represented as mean ± SEM). **c** A significant increase in tdTomato labeling was also observed in the PFC of ChAT-cre p75^in/in^ animals compared to controls, which reverted back to control levels upon injection of p75ICD (one-way ANOVA, *p < 0.05, **p < 0.01; data are represented as mean ± SEM)

As cBF neurons can regulate neurotransmission by both volume release and synaptic release^2^, we next investigated whether p75^NTR^ expression regulates synaptic connections between cBF neurons and the mPFC. This was achieved by using an anterograde trans-synaptic Herpes simplex virus (HSV129; Supplementary Figure S3a) to label the postsynaptic neurons innervated by cholinergic and/or non-cholinergic basal forebrain synaptic efferents^30^. This virus expresses GFP in non-cre recombinase-expressing (non-cholinergic) cells, but undergoes recombination in the cre recombinase-expressing cholinergic cells, leading to the expression of a TdTomato reporter. The native or recombined virus is then passed trans-neuronally to postsynaptic neurons. Viral transmission was limited by sacrificing the animals 72 h after viral injection^36^, and the number of HSV129-labeled cells in the mPFC and hippocampus were counted. The number of TdTomato-expressing cells in the mPFC of mutant animals was significantly (~10-fold) higher than that found in control ChAT-cre animals (Fig. 3a–c), indicating that the number of synapses per cholinergic basal forebrain axon or neuron was significantly increased.

Interestingly, this hyperconnectivity was not specific to cBF-innervated cells, but was also evident for non-cholinergic innervation, with mutant animals having a significantly higher number of GFP-expressing cells in the mPFC than that observed in ChAT-cre control mice (Fig. 3a, b). Although the ChAT-positive cells in the basal forebrain were TdTomato-positive (Supplementary Figure S3b), a minority of cells were both TdTomato- and GFP-positive (Supplementary Figure S3c). However, about half of the TdTomato-labeled cells in the mPFC were also labeled by GFP, and this proportion did not change with genotype (Supplementary Figure S3d). These results suggest that p75^NTR^ affects the synaptic connectivity of cholinergic neurons to the mPFC, and also indirectly influences non-cholinergic afferent synaptic innervation of neurons within this region.

In contrast to these results, no difference in the extent of either cholinergic or non-cholinergic innervation was seen in the hippocampus of the same animals, although many fewer rostral hippocampal neurons were labeled by the HSV129 virus than mPFC cells, despite the former being a larger target structure (Supplementary Figure S3e–g). These data suggest that the role of p75^NTR^ and the nature of cBF neuronal innervation of postsynaptic neurons varies between target brain areas.

To determine whether restoring p75^NTR^ expression affected the synaptic hyperconnectivity, we injected the HSV129 virus into ChAT-cre p75^in/in^ and ChAT-cre control animals that had been previously injected with the p75ICD-GFP AAV. The expression of p75ICD-GFP had no significant effect on the extent of septohippocampal innervation in either case (Supplementary Figure S3e–g). However, there were significantly fewer TdTomato-labeled cholinergic cells in the mPFC of mutant animals in which the p75ICD-GFP was expressed compared to the number in ChAT-cre p75^in/in^ animals injected with the control Tau-GFP AAV (Fig. 3a, c). Non-cholinergic synaptic innervation to the PFC was also significantly reduced in p75ICD-expressing mutant animals (Fig. 3e–g). The levels of both cholinergic and non-cholinergic synaptic innervation in the ChAT-cre p75^in/in^ + p75ICD-GFP animals were not significantly different from those in ChAT-cre control mice. This indicates that re-introduction of p75^NTR^ to adult cBF neurons restores mPFC synaptic connectivity back to wild-type levels, but does not affect septohippocampal innervation.

### Basal forebrain synaptic hyperinnervation to the PFC reduces postsynaptic activity within the mPFC

**Fig. 4:**
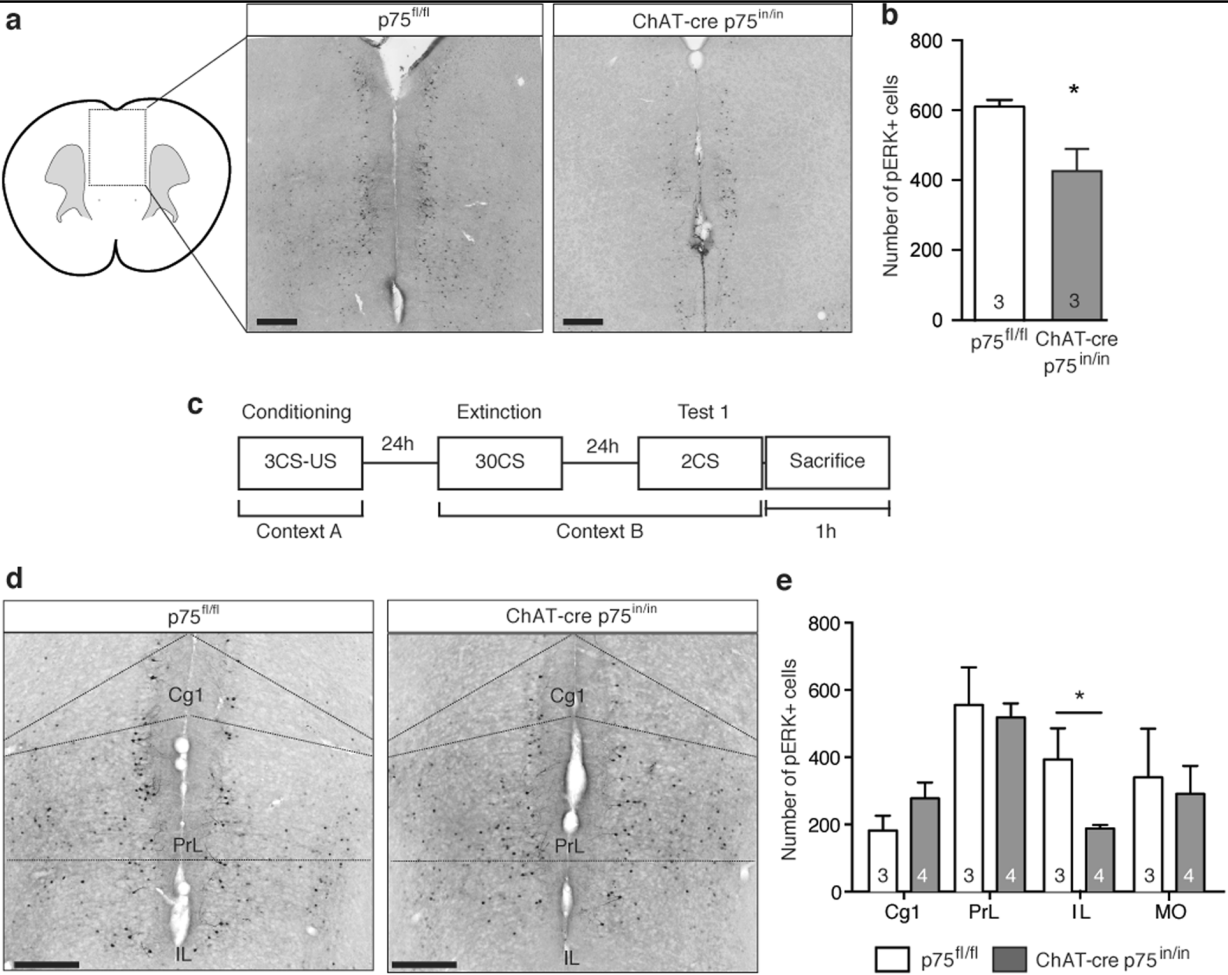
p75^NTR^ affects behaviourally relevant neuronal activity. **a** Representative images of the prefrontal cortex (PFC) of control and mutant mice. Activated cells are visualized with phosphorylated extracellular signal-regulated kinase (pERK). Scale bar = 400 µm. **b** A significant decrease in the number of pERK-positive cells was observed in the mPFC of naïve mutant animals compared to controls. **c** Animals were euthanized within one hour of Test 1 to analyze cells that were activated during the fear extinction consolidation test. **d** Representative images of the prefrontal cortex of extinction-tested control and mutant mice, immunostained for pERK. Scale bar = 400 µm. **e** A significant decrease in the number of pERK-positive cells was observed specifically within the IL of mutant animals (*t* test; *p < 0.05; data are represented as mean ± SEM; The number of animals analyzed is indicated in the graphs); Cg1 cingulate cortex, PrL prelimbic prefrontal cortex, IL infralimbic prefrontal cortex

We next asked whether postsynaptic neuronal activity was altered in the mPFC of p75^NTR^-deficient mice. The activation of the calcium-activated signaling pathway via the phosphorylated extracellular signal-regulated kinase (pERK) was assessed as pERK activity in neurons is observed immediately following cellular activity^37^. The number of pERK-positive cells in the mPFC was first determined for naïve p75^fl/fl^ and ChAT-cre p75^in/in^ animals, revealing a significant reduction in the p75^NTR^-deficient animals compared to controls (Fig. 4a, b). To explore whether the ERK activity correlated with neuronal function, the number of pERK-positive cells in mPFC was determined for mice euthanized within 1 h of fear extinction recall (Fig. 4c). The number of pERK-positive cells within the mPFC was significantly increased compared to that of naïve mice. Furthermore, although the number of pERK cels in the prelimbic, cingulate and medial orbital cortices did not differ between the two genotypes (Fig. 4d–f), a significant decrease in the number of pERK-positive cells within the ILPFC, a nucleus of the mPFC that is critically involved in fear extinction consolidation^38^, was observed in the ChAT-cre p75^in/in^ animals compared to the p75^fl/fl^ controls (Fig. 4d–f).

In order to determine which types of cells were activated in the mutant and control mice, we co-stained mPFC sections for pERK and GABAergic, glutamatergic or cholecystokinin (CCK) markers, namely parvalbumin, calcium/calmodulin-dependent protein kinase II (CaMKII) and CCK antibodies, respectively. pERK did not colocalize with either GABAergic or CCK markers (data not shown), whereas ~15% of pERK-positive cells in the ILPFC of mice of both genotypes co-stained with the glutamatergic marker (Supplementary Figure S4a, b). As there were 50% fewer pERK-positive cells in the ILPFC after extinction testing, these data indicate that the cBF synaptic hyperinnervation of the mPFC, which arises due to the loss of p75^NTR^ expression, reduces by half the number of principal glutamatergic neurons within the ILPFC that are activated following fear extinction recall, potentially explaining the behavioral change in the p75^NTR^-deficient mice.

## Discussion

Here we demonstrate that loss of p75^NTR^ expression from cBF neurons induces synaptic hyperconnectivity within the mPFC and results in altered fear extinction consolidation. Re-expression of a constitutively active fragment of p75^NTR^ in the form of p75ICD-GFP in mPFC-projecting cBF neurons of adult ChAT-cre p75^in/in^ mice not only restored fear extinction behavior, but also reversed the level of basal forebrain connectivity in the mPFC to that observed in control animals. These results indicate that adult p75^NTR^ signaling is important for regulating connectivity between basal forebrain and mPFC neurons, which in turn modulates fear extinction consolidation.

The major cellular phenotype of cBF neurons observed in ChAT-cre p75^in/in^ mice was a ~10-fold increase in efferent connectivity to the mPFC, as measured by trans-neuronal tracing. Interestingly, we did not find a corresponding increase in the axonal arborization of individual neurons projecting to either the mPFC or the hippocampus. Although an increase in the density of hippocampal innervation in a complete p75^NTR^-deficient mouse has been reported^18,19^, our previous study using conditional ChAT-cre p75^in/in^ mice did not reproduce this, instead finding a significant increase in cBF axonal density specifically to layer V neurons in the barrel cortex^16^. Given that the methods used in the previous studies did not account for the 25% increase in cBF neuronal number, our current findings suggest that p75^NTR^ does not play a substantive role in regulating the extent of axonal arborization of individual cBF neurons, but rather regulates the number of synaptic connections that each cBF axon makes with its target(s). This would support the notion that the previous observations were a consequence of an increased number of neurons rather than changes to individual arbor sizes. In support of this interpretation, the expression of p75ICD-GFP had no discernable pruning effect on overall axonal innervation but reversed the synaptic hyperinnervation in the mPFC of mutant mice without affecting cBF neuron number. Although we can not rule out a subtle effect on axonal outgrowth due to p75^NTR^, a possible increase in axonal arbor size following p75ICD expression correlates with reduced cBF synapse density and therefore cannot be the explanation for our results.

The observed changes to the synaptic innervation patterns of basal forebrain neurons in the mPFC of ChAT-cre p75^in/in^ animals could be due to mutant cells having increased release of the HSV129 virus, resulting in increased expression of postsynaptic HSV129 markers in these mice. However, this is unlikely given that the number of postsynaptic hippocampal cells labeled by HSV129 was not significantly different between control and knockout animals of the same cohort (Supplementary Figure S3e–g). Furthermore, we found a change in innervation patterns of both cholinergic and non-cholinergic targets in the mPFC that could not be fully accounted for by local basal forebrain connectivity. Taken together, these findings suggest that the observed cellular phenotypes in mutant mice are not due to differences in the uptake or release of HSV129. Rather the observed changes to postsynaptic innervation in the mutant mice are specific to mPFC-projecting cBF neurons due to the absence of p75^NTR^.

We were surprised by the finding that non-cholinergic basal forebrain innervation to the mPFC of p75^NTR^-deficient mice was altered, given that p75^NTR^ is only expressed by, and was genetically eliminated from, cBF neurons. However, the rescue of this phenotype by re-expression of p75ICD-GFP in ChAT-cre p75^in/in^ mice indicates that the increase in non-cholinergic synaptic innervation was mediated by a cholinergic mechanism. Our tracing studies revealed that ~50% of basal forebrain postsynaptic targets in the mPFC were co-labeled by both recombined HSV129 (derived from a cre-positive cell) and native HSV129 (derived from a cre-negative cell), suggesting that a significant proportion of mPFC neurons receive input from both cholinergic and non-cholinergic basal forebrain neurons. Analysis of co-expression in the basal forebrain revealed that a subset of tdTomato-positive cells co-expressed GFP, and expression at 72 h was not limited to ChAT-positive cells, suggestive of innervation of neighboring cells by cBF neurons. It has previously been shown that there is cross-talk between cell populations in the basal forebrain, with the activity of GABAergic cells of the basal forebrain driving the efflux of acetylcholine^39^, and GABA often being co-released with acetylcholine^40^. We therefore postulate that the observed cBF synaptic hyperinnervation in the absence of p75^NTR^ drives the recruitment of additional synaptic input from other cell and neurotransmitter types within the basal forebrain-mPFC circuit, which in turn influences the balance of cortical activity and output.

Despite the strong indication of hyperinnervation in the mPFC of ChAT-cre p75^in/in^ mice, the synaptic innervation in the hippocampus of mutant animals of the same cohort was unchanged, and expression of the p75ICD-GFP had no discernable effect in this region. Furthermore, despite robust labeling of axons by the Tau-GFP virus, there were many fewer HSV129-infected postsynaptic cells in the hippocampus compared to the number found in the mPFC. This suggests that septohippocampal basal forebrain neurons may exert their influence on hippocampal activity by volume rather than synaptic neurotransmission^7,41–43^. This would explain why we failed to observe any changes in septohippocampal neuronal structure or hippocampal function in our previous^16^ or present study. Secondly, our results indicate that p75^NTR^ specifically regulates the point-to-point connectivity of neurons in the basal forebrain to their synaptically connected targets, while having little or no effect in areas where volume neurotransmission may predominate. However, future studies will need to address this further through electrophysiological assessments of hippocampal and cortical activity in the absence of p75^NTR^ in cBF neurons

Basal forebrain hyperinnervation to the mPFC reduced the level of basal neuronal activity in the mPFC as measured by pERK immunostaining. Whether these cells are less synaptically active or other signaling activity is reduced is unclear. However, the decrease in pERK-positive cells specifically within the ILPFC immediately after testing for fear extinction consolidation suggests a change in physiolgically relevant neuronal activity within this cortical region. It is widely accepted that the ILFPC plays a critical role in fear extinction, with activation of ILPFC glutamatergic neurons that project to the central amygdala being a key means by which fear-related responses are inhibited following extinction^24–26,44,45^. Therefore, the observed decrease in glutamatergic neuron pERK activity within the ILPFC of ChAT-cre p75^in/in^ animals is consistent with the observed deficit in extinction consolidation in the mutant mice.

The expression of p75ICD-GFP within the medial septum cBF neurons of the p75^NTR^-deficient mice both reversed the mPFC synaptic hyperinnervation and rescued fear extinction behavior. These data strongly link the p75^NTR^-mediated synaptic changes within the fear extinction circuit to the observed behavioral change. Several other reports suggest that loss of p75^NTR^ can affect the synaptic function of neurons both directly and indirectly^3,17,46,47^. Our study cannot resolve whether basal forebrain hyperinnervation of the ILPFC directly underlies reduced ILPFC neuronal activity, or whether hyperinnervation to other mPFC nuclei and/or other cortical areas indirectly influences ILPFC activity during fear extinction consolidation and testing. For example, we have previously demonstrated that ChAT-cre p75^in/in^ mice have better spatial memory in an allothetic (uncued) place avoidance spatial memory task^16^. Therefore, it is possible that the altered extinction is due to enhanced recall of the CS-US pairing^43^. Similarly, acetylcholine is released during associative learning^2^, and the enhanced fear recall in the p75^NTR^-deficient mice could be due to altered context-dependent associations. Regardless of the mechanism, our study provides compelling evidence that successful consolidation of fear extinction memories requires coordinated activity between cholinergic and non-cholinergic neurons in the basal forebrain and glutamatergic neurons within the mPFC, and demonstrates that this process is controlled by p75^NTR^-mediated signaling.

In summary, this work has highlighted that the nature of cBF neuronal innervation of postsynaptic neurons varies between target areas and has revealed that cBF synaptic connectivity in the adult is regulated by p75^NTR^ signaling. We have further determined a role for medial septal cBF neurons projecting to the mPFC in the regulation of fear extinction. As such, cholinergic or neurotrophic modulators could be considered as candidate therapeutic targets for enhancing memory or, alternatively, for the treatment of conditions such as post-traumatic stress disorder.

## Electronic supplementary material

Supplemental material
