# Supplementary material for: Cholinergic basal forebrain neurons regulate fear extinction consolidation through p75 neurotrophin receptor signaling
Source: Transl Psychiatry. 2018 Sep 21;8:199. doi: 10.1038/s41398-018-0248-x (PMC6154972; doi:10.1038/s41398-018-0248-x)
Supplement: Supplementary file 1 — Supplemental material [file 41398_2018_248_MOESM1_ESM.pdf]

Supplementary figures

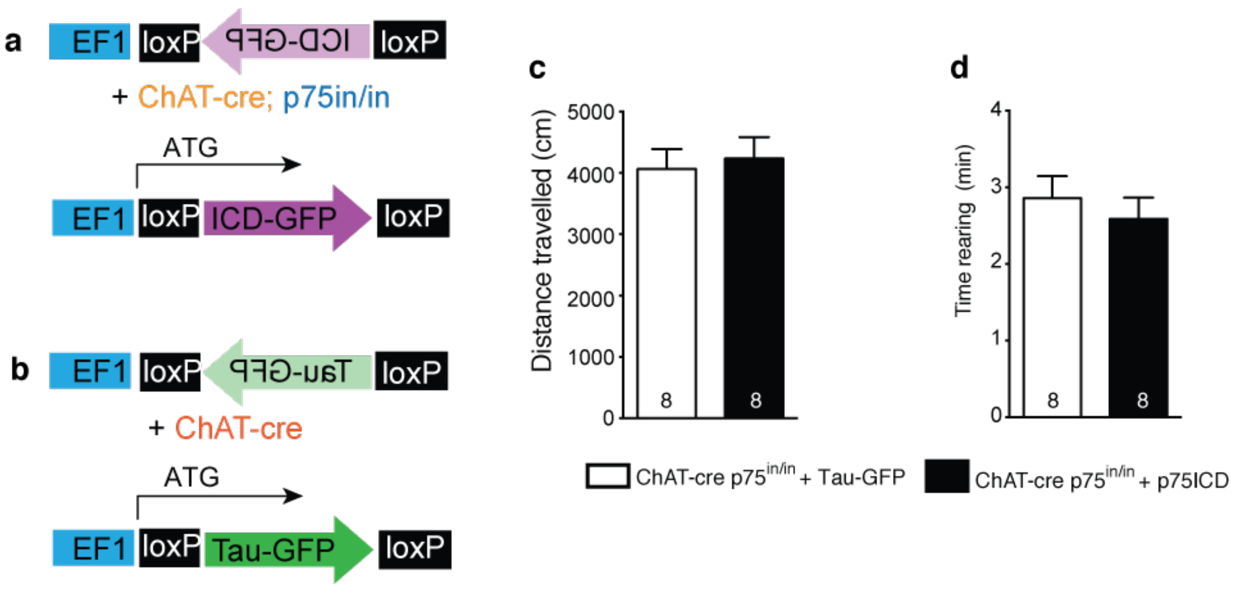

Figure S1.

Schematic of the AAV constructs that contained the (a) p75ICD-GFP and (b) Tau-GFP expression cassettes. The expression cassete is inverted and flanked between two loxP flex sites. When expressed in cre-recombinase-expressing cells, the viral cassete is inverted back to the correct orientation and expressed.

(c) Average distance travelled during an open field test. No significant differences were observed between mutant and control mice injected with p75ICD-GFP, indicating no changes in locomotor behavior in the presence of the virus.

(d) Average rearing time during an open field test. No significant differences were observed between the two groups injected with p75ICD-GFP, indicating no changes to general exploratory behavior in the presence of the virus

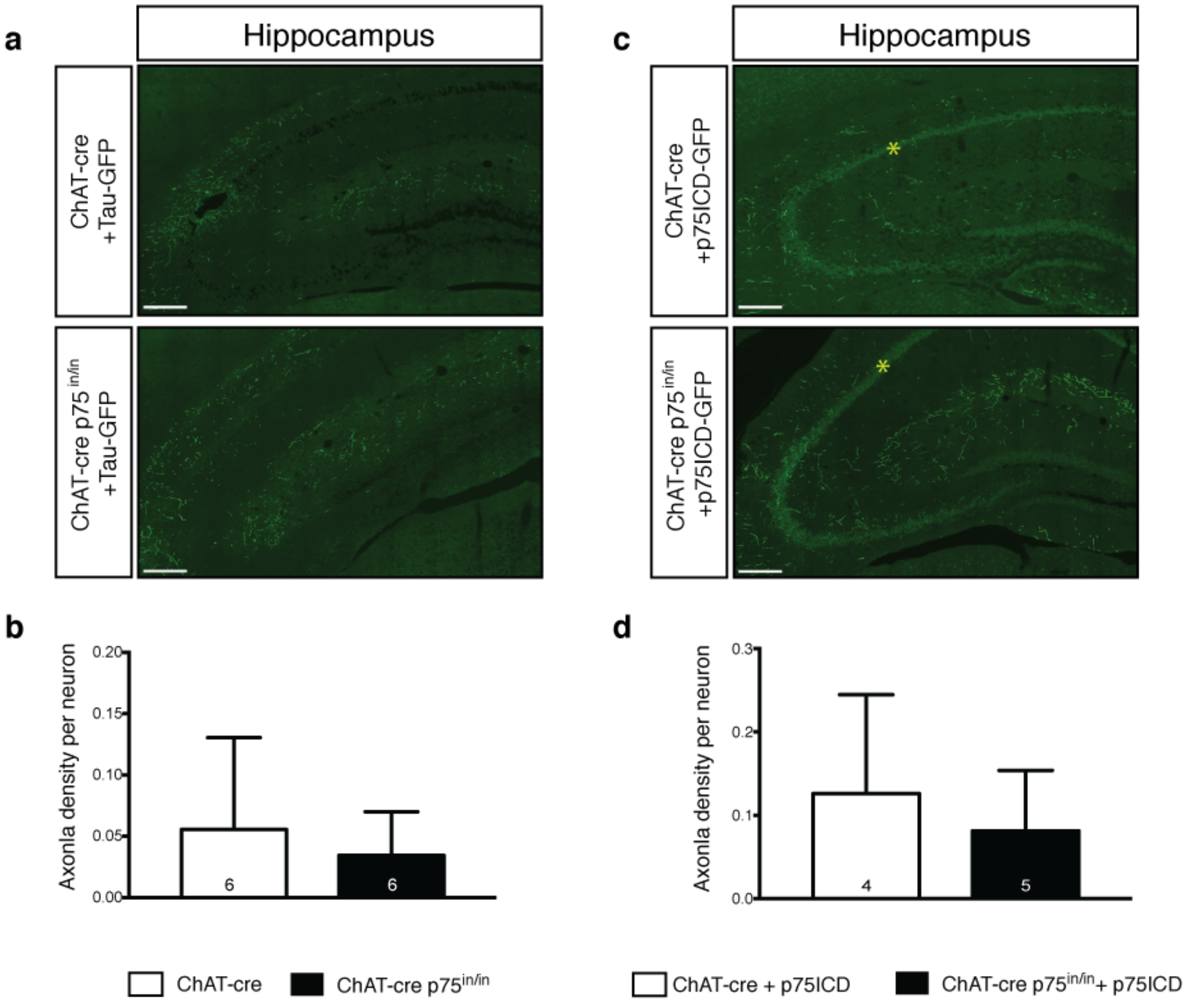

Figure S2.

(a) Representative images of the hippocampus of mutant and control mice following injection of Tau-GFP in the medial septum. Sections were labelled using an anti-GFP antibody (green). Scale bar=200μm.

(b) Average axonal density per infected neuron for animals injected with Tau-GFP. No significant differences in axonal innervation was observed between the two groups.

(c) Representative images of the hippocampus of mutant and control mice following injection of p75ICD-GFP in the medial septum. Sections were labelled using an anti-GFP antibody (green). Scale bar=200μm. * indicates autofluorescence in the pyramidal neurons of the hippocampus in these sections.

(d) Average axonal density per infected neuron for animals of animals injected with p75ICD-GFP. No significant differences were observed between the two groups.

The number of animals analyzed is indicated in the graphs.

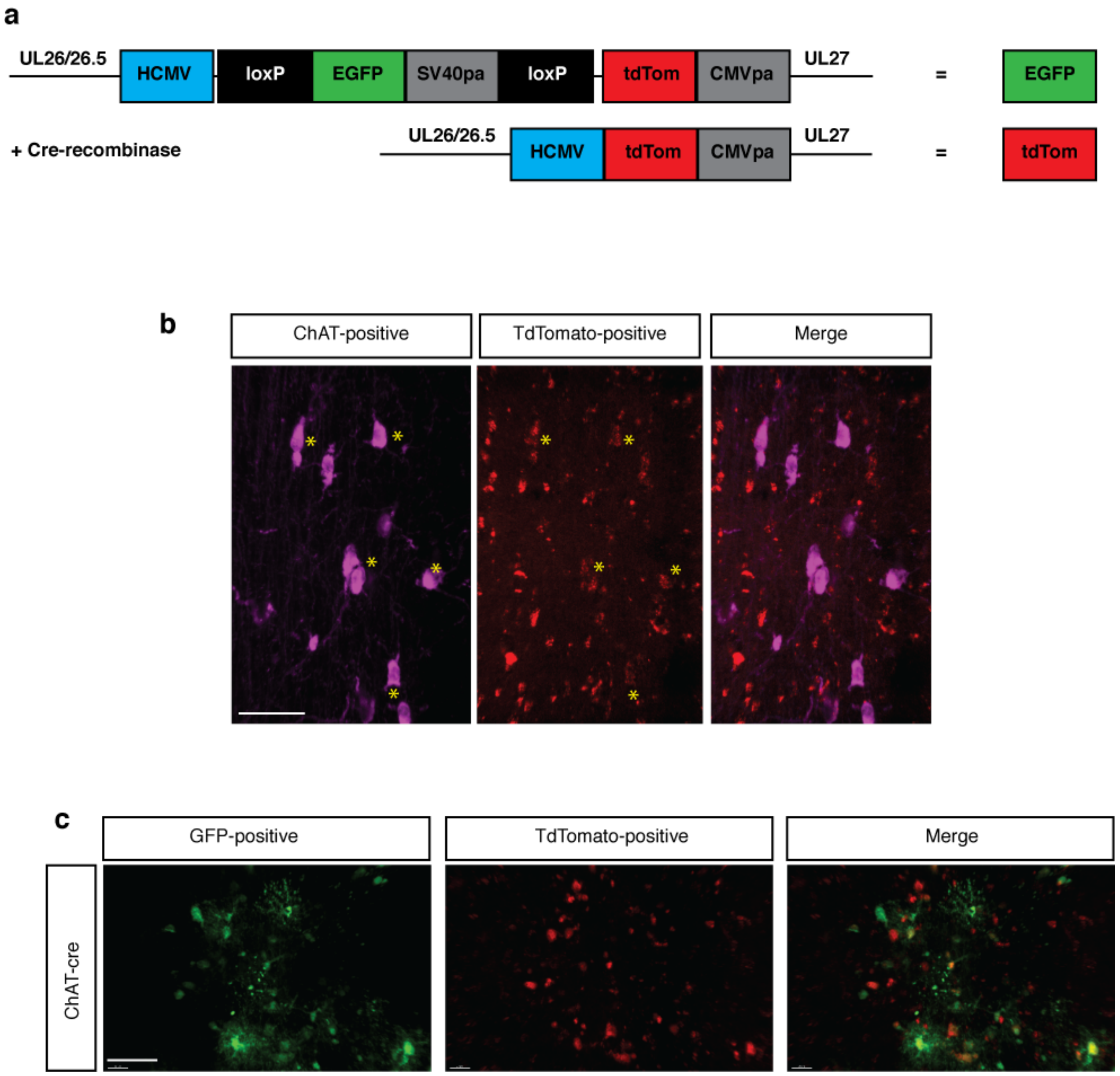

Figure S3.

(a) Schematic of the HSV129 virus (adapted from McGovern et al., 2015). The virus expresses GFP in non-cre recombinase-expressing cells. In the presence of cre recombinase, the GFP cassete flanked by the loxP sites is excised, leading to expression of the tdTomato reporter.

(b) Photographs of the basal forebrain 72 hours after HSV129 injection, stained for ChAT (purple) and TdTomato (red). Yellow stars mark cells co-expressing GFP and tdTomato cells.

(c) Photographs of basal forebrain 48 hours after HSV192 injection immunostained for TdTomato and GFP. Yellow indicates co-expression of GFP and tdTomato cells.

Scale bars =50μm

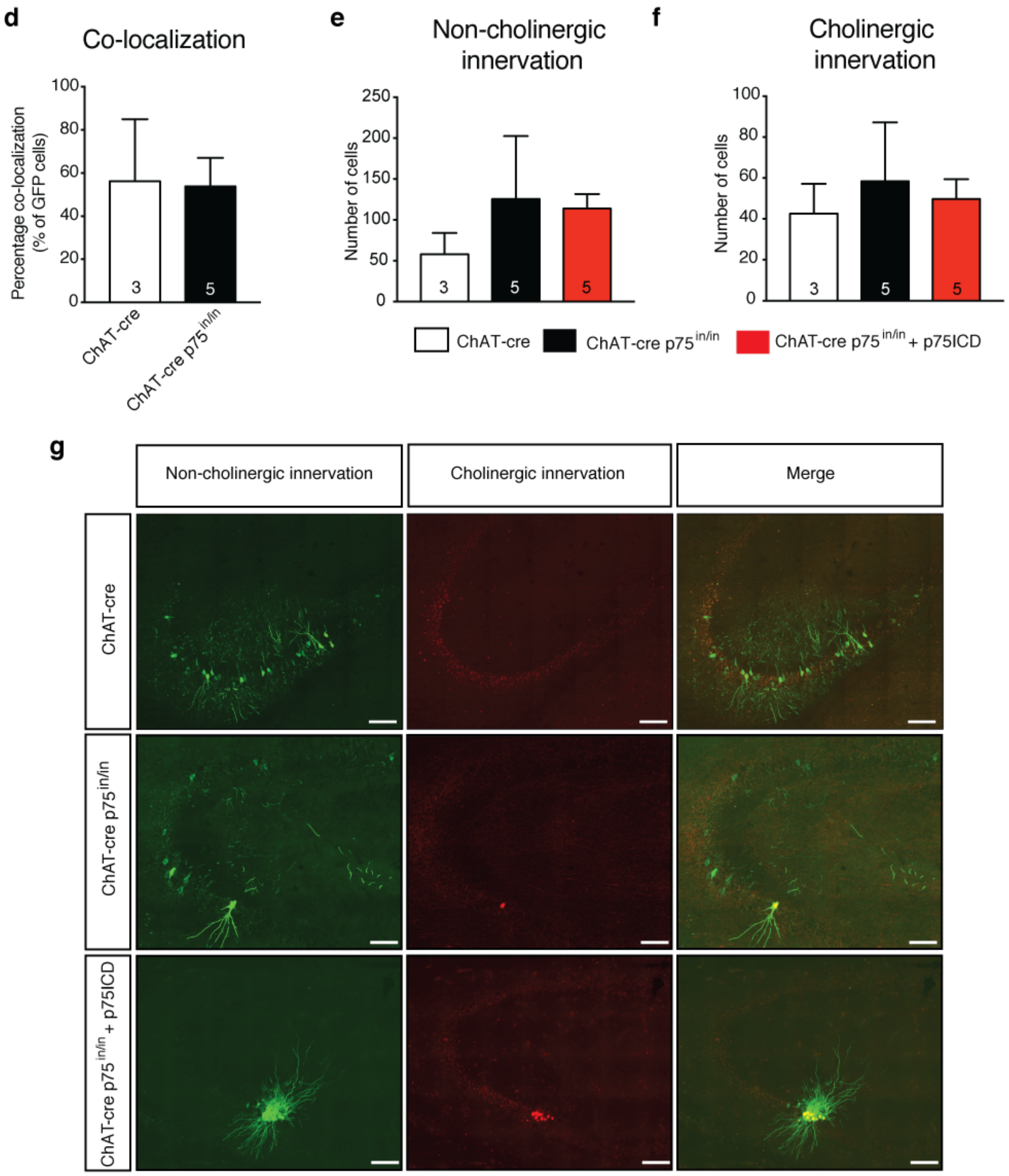

Figure S3 continued.

(d) Average percentage of cells in the PFC that showed expression of both the GFP and tdTomato markers. An average of ~50% of cells in both mutant and control mice showed innervation by both cholinergic and non-cholinergic cells.

(e) There were no significant differences in GFP labeling in the hippocampus between the two groups.

(f) There were no significant differences in tdTomato labeling in the hippocampus between the two groups.

(e) Representative images of the hippocampus of cre-expressing controls (ChAT-cre), mutants (ChAT-cre $p75^{in/in}$) and mutants injected with p75ICD-GFP. Postsynaptic cells in the hippocampus innervated by non-cholinergic basal forebrain neurons (green) were labeled against the GFP-expressing version of HSV129 virus. Cells innervated by cholinergic basal forebrain neurons (red) were labeled against the tdTomato-expressing version of the HSV129 virus.

Scale bar=100μm. The number of animals analyzed is indicated in the graphs

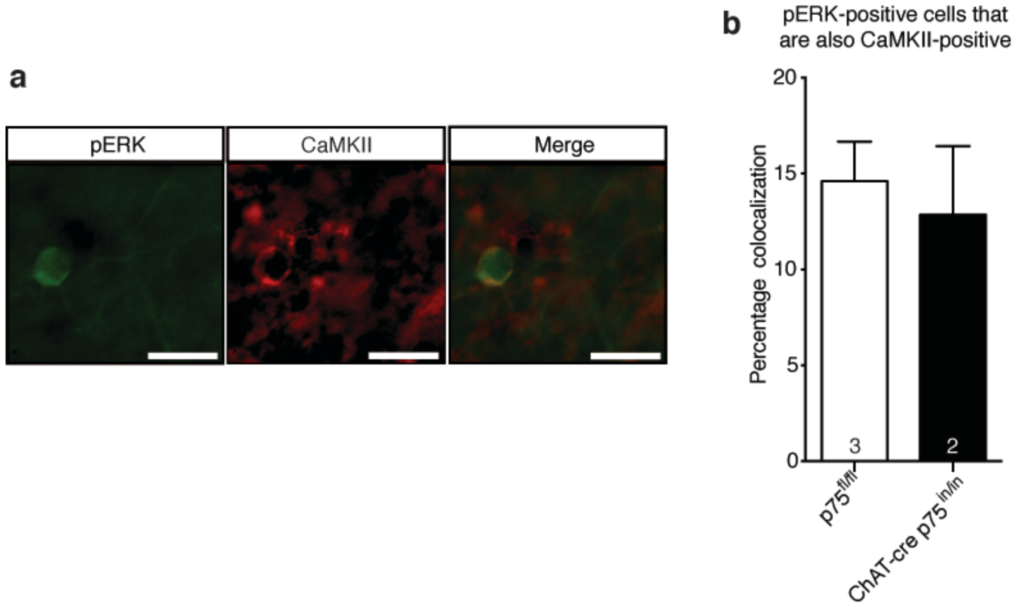

Figure S4.

(a) Representative image of a subpopulation of pERK-activated cells (red) that also expressed the glutamatergic marker Ca^{2+}/calmodulin-dependent protein kinase II (CaMKII; red). Scale bar=50μm.

(b) Average percentage of glutamatergic cells labeled with CaMKII that colocalized with pERK. No significant difference in the percentage of activated glutamatergic cells was observed between the two groups. As there were 50% fewer pERK-postiive cells in the infralimbic prefrontal cortex (Figure 4c), this result suggests that signficantly fewer glutamatergic cells were activated in the mutant animals during the fear extinction test. The number of animals analyzed is indicated on the graph.
